# Status and gaps in newborn hearing screening in South Africa: A cross-sectoral review

**DOI:** 10.4102/sajcd.v73i1.1165

**Published:** 2026-05-30

**Authors:** Karin Joubert, Duoné Swart

**Affiliations:** 1 Department of Audiology, Faculty of Humanities, University of the Witwatersrand, Johannesburg, South Africa

**Keywords:** newborn hearing screening, South Africa, audiology, early hearing detection, task-shifting

## Abstract

**Background:**

Newborn hearing screening (NHS) is recognised as a key component of early hearing detection and intervention (EHDI). In South Africa, implementation remains inconsistent across provinces and health sectors, with limited national coordination and policy support. Updated data are needed to identify progress and persisting gaps in service delivery.

**Objectives:**

To describe the status of NHS in South Africa across public, private and non-governmental (NGO) sectors, focusing on the availability of services, equipment and personnel, and to identify barriers to implementation.

**Method:**

A cross-sectional, descriptive online survey was distributed to audiologists practising in South Africa using REDCap (Research Electronic Data Capture). A total of 133 audiologists participated, representing all nine provinces and the public (52%), private (40%) and NGO (6%) sectors.

**Results:**

Newborn hearing screening services were concentrated in urban provinces, mainly Gauteng and the Western Cape. The public sector accounted for 49% of services, largely at tertiary hospitals. Equipment availability was generally high. mHealth adoption was limited (4% in public sector only). Audiologists conducted most screenings, although task-shifting to trained non-audiologists occurred in NGOs. Major barriers included limited parental awareness, poor follow-up, staff shortages, funding constraints and a lack of medical aid coverage in the private sector.

**Conclusion:**

Despite progress since the 2008 baseline, when only 7.5% of public hospitals offered NHS, coverage remains uneven, with persistent geographic and resource inequities. Integrating NHS into primary healthcare, expanding task-shifting and improving public awareness are essential for equitable access.

**Contribution:**

This study provides the most recent cross-sectoral overview of NHS in South Africa, informing policy and supporting equitable EHDI scale-up aligned with World Health Organization (WHO) targets.

## Introduction

Globally, disabling hearing impairment is considered one of the most prevalent disabilities (World Health Organisation [WHO], 2021a). Over 430 million people worldwide experience disabling hearing loss, including more than 34 million children (WHO, 2021b). In the paediatric population, auditory input plays a critical role in speech, language and cognitive development (HPCSA, 2018). There is a limited neurobiological window during early childhood in which hearing supports speech and language acquisition (Sharma et al., 2009). If hearing loss is undetected or unmanaged during this sensitive period, it may result in long-term developmental delays (Al-Ani, 2023; Sharma et al., 2009). Early detection and timely intervention are therefore essential to mitigating the impact of childhood hearing loss. Universal newborn hearing screening (UNHS) is endorsed by the WHO as the preferred secondary prevention strategy (WHO, 2021a, 2021b). This proactive approach involves screening all newborns shortly after birth for potential hearing loss, regardless of known risk factors (Kanji, 2021a; Olusanya et al., 2014). Internationally, the Joint Committee on Infant Hearing (JCIH) established the 1-3-6 principle as the gold standard for early hearing detection and intervention (EHDI) programmes, recommending that hearing screening occur before 1 month of age, diagnostic evaluation before 3 months, and enrolment in early intervention before 6 months (JCIH, 2019). Recognising the structural constraints of South Africa’s healthcare system and the integration of maternal and child health services with routine immunisation schedules, the Health Professions Council of South Africa (HPCSA) established adapted timeframes. The HPCSA EHDI guideline recommends initial screening by 6 weeks of age (aligned with the first immunisation visit), confirmation of hearing loss by 4 months, and intervention initiation before 8 months (HPCSA, 2018). The HPCSA (2018) further specifies a bilateral pass criterion, requiring infants to obtain a pass result in both ears during the same screening session; infants who do not meet this criterion require outpatient rescreening within 1–2 weeks, with diagnostic evaluation at district or tertiary-level hospitals if the refer result persists. Ideally, screening programmes should detect both bilateral and unilateral hearing losses. However, where limited resources prevent this, programmes may initially employ protocols targeting bilateral loss only, with the intention of expanding to unilateral loss detection as capacity develops (HPCSA, 2018). This guideline further proposes two implementation pathways for newborn hearing screening (NHS): hospital-based screening prior to discharge from neonatal units (including well-baby nurseries, intensive care, high care and kangaroo mother care wards), and community-based screening at primary healthcare (PHC) clinics and midwife obstetric units (MOUs) (HPCSA, 2018). Newborn hearing screening screening employs two primary physiological technologies as the gold standard: Otoacoustic emissions (OAE) and automated auditory brainstem response (AABR) (HPCSA, 2018; Madzivhandila et al., 2024; Wen et al., 2022). Otoacoustic emissions measures cochlear outer hair cell function via a probe placed in the ear canal. This method is characterised by its simplicity, speed and relatively low cost, but is susceptible to false positives from middle ear fluid and vernix in the ear canal, particularly when conducted within 48 h of birth (Sheng et al., 2021). Automated auditory brainstem response evaluates the entire auditory pathway, including the auditory nerve and brainstem, via electrodes placed on the scalp (Sheng et al., 2021). Automated auditory brainstem response provides superior detection of auditory neuropathy with lower false-positive rates, but can be time-consuming and costly (Wen et al., 2022). The choices between these technologies and screening platforms involve balancing sensitivity, specificity, feasibility and resource availability (Kanji, 2021a; Sheng et al., 2021; Wen et al., 2022). Evidence from high-income countries has shown that well-implemented UNHS programmes improve language, cognitive, academic and psychosocial outcomes for children with early-identified hearing loss (WHO, 2021b). Despite these benefits, South Africa does not mandate UNHS as part of its national health policy. The country faces competing health priorities known as the quadruple burden of disease, which include high rates of human immunodeficiency virus (HIV) and tuberculosis, maternal and child mortality, violence and injuries, and rising non-communicable diseases (Basu, 2018). These systemic pressures limit the prioritisation of preventative services such as NHS. Nevertheless, short-term and pilot NHS programmes have been implemented in select provinces, largely driven by individual hospitals, research initiatives, or non-governmental organisations (Kanji, 2021b; Khoza-Shangase & Harbinson, 2015). South Africa’s healthcare system is two-tiered, comprising a publicly funded sector that serves approximately 84.2% of the population, and a private sector accessible to the 15.8% with private health insurance (Cowling, 2023). While the public sector is often under-resourced and overburdened, there has been recent growth in the NHS within the private sector. A 2011 national survey found only 14% of private maternity units offered UNHS (Meyer & Swanepoel, 2011). However, by 2019, Netcare, one of South Africa’s largest private healthcare providers, had launched a nationwide NHS programme across maternity units (Netcare Limited, 2023a). By June 2022, the programme had screened 69 787 babies (Netcare Limited, 2023a) with an estimated coverage rate of 76.43%. The coverage rate was estimated using indicators reported by Netcare (Netcare Limited 2020, 2021, 2022, 2023b). In contrast, NHS in the public sector remains limited. A 2008 national survey found that only 7.5% of public hospitals offered some form of NHS, with fewer than 1% providing UNHS (Theunissen & Swanepoel, 2008). Since then, several feasibility studies have explored the implementation of NHS at various levels of care, including primary healthcare facilities, maternity and obstetric units, and hospitals (De Kock et al., 2016; Friderichs et al., 2012; Kgare & Joubert, 2024; Khoza-Shangase et al., 2018; Khoza-Shangase & Harbinson, 2015). More recently, a national public health survey by Bhamjee et al. (2022) found that 51% of public hospitals offered some form of NHS (Bhamjee et al., 2022), with the majority conducting targeted, risk-based screening. These findings indicate incremental progress, but NHS coverage in the public sector remains limited and inconsistent, with most programmes falling short of international UNHS benchmarks. Furthermore, these efforts remain fragmented, and South Africa continues to lack a nationally coordinated or mandated NHS programme (Kanji, 2021b). Several barriers hinder the implementation and scaling of NHS in South Africa. These include limited budgets, lack of screening equipment, uncalibrated or broken devices, and a shortage of audiologists (Bhamjee et al., 2022; Khoza-Shangase, 2021; Naidoo & Khan, 2022). The disparity of audiologists between urban and rural areas, and across provinces, further exacerbates access inequities (Pillay et al., 2020). Public sector audiology posts are also limited because of budgetary constraints and austerity measures, which further impede service delivery (Khoza-Shangase, 2025). To address these challenges, task-shifting models have been proposed. Such models have shown promise in other low- and middle-income countries (LMICs) and are supported by WHO recommendations for integrated ear and hearing care (Eksteen et al., 2019; Suen et al., 2019; WHO, 2021a). Importantly, task-shifting in NHS has been shown to be feasible and acceptable when adequate training and supervision are provided (Bright et al., 2019; Olusanya et al., 2014). Although some progress has been made in both the public and private sectors, the most recent national surveys assessing NHS practices in South Africa were conducted independently within specific sectors. As a result, there is a lack of cross-sectoral reviews that provide a comprehensive understanding of NHS implementation across the health system. Updated, integrated data are therefore essential for informing national policy development, health system planning and the sustainable implementation of NHS programmes. The aim of this study was thus to describe the status of NHS in South Africa, focusing on the availability of services, equipment and personnel, as well as the challenges faced, and reasons for non-implementation across the public, private and non-governmental sectors.

## Research methods and design

### Participants

Participants were audiologists practising in South Africa, recruited through two professional organisations which distributed an invitation and survey link to their respective membership databases. A total of 133 completed surveys were included in the analysis. The majority of respondents were employed in the public health sector (52%; *n* = 68), followed by the private sector (40%; *n* = 53) and non-governmental organisations (NGOs) (6%; *n* = 8). The remaining participants were employed in academic or corporate settings. Among those working in the public sector (*n* = 68), 46% (*n* = 32) were based at tertiary hospitals, 28% (*n* = 19) at district hospitals, 16% (*n* = 11) at regional hospitals, 4% (*n* = 3) at community health clinics and 3% (*n* = 2) at primary healthcare clinics. Participants had a mean of 8.74 years of clinical experience (standard deviation [s.d.] = 7.94; range: 1–35 years).

### Materials

A self-developed, descriptive survey consisting of 36 questions across three sections was used to collect data. The survey was developed based on WHO recommendations for early hearing detection and intervention programmes (WHO, 2020, 2021b) and existing literature on barriers to NHS implementation in low- and middle-income countries (El-Jardali et al., 2019; Kanji, 2021a, 2022; Kgare & Joubert, 2024; Naidoo & Khan, 2022). The questionnaire included a mix of closed-ended, open-ended and Likert-type questions. The first section gathered demographic information and details about respondents’ clinical experience. The subsequent sections were tailored based on whether participants conducted hearing screenings. For those who performed hearing screenings, the survey explored aspects such as the equipment used, personnel involved, screening sites, recruitment and training of screeners, and overall programme management. For those not involved in hearing screening, the survey examined referral practices and perceived barriers to conducting screenings. The survey underwent a pilot testing phase with three audiologists to assess face validity, item clarity and completion time. Feedback from pilot participants resulted in the rewording of three questions for clarity and the addition of a question on the use of mHealth technology.

### Procedures

Data were collected between March 2022 and July 2022. The survey was distributed using REDCap (Research Electronic Data Capture), a secure, web-based platform designed to support data capture for research studies. The software is licensed by the University of the Witwatersrand. Potential participants received an email from their professional organisation containing a link to the survey, along with a participant information sheet detailing the purpose and nature of the study. At the beginning of the survey, participants were assured of anonymity and confidentiality and were informed that completion of the survey implied consent.

### Statistical analysis

Survey data were exported from REDCap into Microsoft^®^ Excel^®^ (for Microsoft 365 MSO – Version 2602) for analysis. Descriptive statistics were used to summarise and analyse the data.

### Ethical considerations

This project was approved by the University of the Witwatersrand Human Research Ethics Committee (Non-medical) (Protocol number: H211015) prior to data collection.

## Results

### Availability of newborn hearing screening services and equipment

The availability of NHS services varied considerably across South African provinces and healthcare sectors (Table 1). Of the 108 reported NHS services, 49% (*n* = 53) were provided in the public sector, 44% (*n* = 48) in the private sector and 7% (*n* = 7) by non-governmental organisations (NGOs). Gauteng and the Western Cape accounted for the majority of services, contributing 32% (*n* = 35) and 24% (*n* = 26), respectively. These two provinces also demonstrated the greatest distribution across all three sectors. In contrast, provinces such as North West (1%), Limpopo (5%) and Mpumalanga (6%) reported fewer services, with several provinces lacking services in the private and/or NGO sectors. Notably, private sector NHS services were absent in Limpopo, Mpumalanga and North West, while NGO involvement was limited to four provinces.

**TABLE 1:** Availability of newborn hearing screening services per province and health sector (*N* = 108).

Province	Private (*n* = 48)		Public (*n* = 53)		NGO (*n* = 7)		Total	
	*n*	%	*n*	%	*n*	%	*n*	%
Eastern Cape	5	10	7	13	0	0	12	11
Free State	5	10	3	6	1	14	9	8
Gauteng	19	40	13	25	3	43	35	32
KwaZulu-Natal	6	13	2	4	0	0	8	7
Limpopo	0	0	4	8	1	14	5	5
Mpumalanga	0	0	6	11	0	0	6	6
Northern Cape	2	4	4	8	0	0	6	6
North West	0	0	1	2	0	0	1	1
Western Cape	11	23	13	25	2	29	26	24

**Total**	**48**	**100**	**53**	**100**	**7**	**100**	**108**	**100**

NGO, non-governmental organisation; NHS, newborn hearing screening.

In the public sector, hearing screening is most commonly offered at tertiary-level hospitals (55%; *n* = 31), followed by district hospitals (21%; *n* = 12) and regional hospitals (16%; *n* = 9). Screening services are limited at community-based facilities, with only 4% (*n* = 2) available at primary healthcare centres and 4% (*n* = 2) at community health clinics.

### Availability of newborn hearing screening equipment

Table 2 presents the availability of NHS equipment across the private, public and NGO sectors. The three most commonly used types of equipment, AABR, OAE and tympanometry were similarly distributed across sectors. The public sector reported the highest absolute availability for each (57%; *n* = 30), followed by the private sector (49%; *n* = 23). The NGO sector, while representing a small portion of services, reported the highest relative availability of AABR (86%; *n* = 6), OAE (71%; *n* = 5) and tympanometry (57%; *n* = 4). This suggests that although NGOs operate fewer sites, these are generally well-equipped. Use of mobile health (mHealth) technology for hearing screening was limited to only 4% (*n* = 2) of public sector sites, with no adoption reported in either the private or NGO sectors.

**TABLE 2:** Availability of newborn hearing screening equipment.

Equipment	Private (*n* = 47)		Public (*n* = 53)		NGO (*n* = 7)	
	*n*	%	*n*	%	*n*	%
AABR	23	49	30	57	6	86
Otoacoustic emissions	23	49	30	57	5	71
Tympanometry	23	49	30	57	4	57
mHealth technology	0	0	2	4	0	0

AABR, automated auditory brainstem Rresponse; NGO, non-governmental organisation.

### Personnel involved in newborn hearing screening

Across all healthcare sectors, audiologists are the primary personnel responsible for conducting NHS, with 85% (*n* = 40) of private sector sites, 87% (*n* = 46) of public sector sites and 43% (*n* = 3) of NGO sites reporting audiologist-led screening (Table 3). In total, 18 sites across all sectors utilised non-audiologists for screening, most of whom were trained dedicated hearing screeners (*n* = 14). The number of dedicated screeners per site ranged from one to eight, with a mean of two screeners per site. These personnel received a reported average monthly salary of R5400.00 (~$300.00). Notably, at one public hospital, trained parents of Deaf or hard-of-hearing children were engaged as screeners, while one private practice employed a trained receptionist for this role. In a small number of instances, Speech-Language Therapists conducted the screenings. Importantly, all NHS programmes employing non-audiologist screeners were overseen and managed by audiologists, who were also responsible for the initial training of these individuals.

**TABLE 3:** Newborn hearing screening personnel.

Screening personnel	Private (*n* = 47)		Public (*n* = 53)		NGO (*n* = 7)	
	*n*	%	*n*	%	*n*	%
Audiologists	40	85	46	87	3	43
Non-audiologists	7	15	7	13	4	57

NGO, non-governmental organisation.

### Challenges experienced in newborn hearing screening programmes

Participants across all sectors reported multiple challenges related to the implementation of NHS programmes (Table 4). The two most frequently cited challenges, common to all sectors, were the lack of parental awareness about the importance of hearing screening and poor follow-up attendance after a refer result. These challenges were reported by 28% (*n* = 33) and 22% (*n* = 26) of respondents in the private sector, 16% (*n* = 28) and 21% (*n* = 38) in the public sector, and 19% (*n* = 3) and 25% (*n* = 4) in the NGO sector, respectively.

**TABLE 4:** Challenges experienced in newborn hearing screening programmes.

Challenges	Private (*n* = 118)		Public (*n* = 180)		NGO (*n* = 16)	
	*n*	%	*n*	%	*n*	%
Broken equipment	2	2	20	11	0	0
Equipment not calibrated	1	1	19	11	1	6
Staff shortages	4	3	28	16	3	19
Time constraints	16	14	29	16	2	13
Screening not funded by medical aid	21	18	0	0	2	13
Babies are not brought for screening	15	13	12	7	1	6
Parents do not honour follow-up screening appointments	26	22	38	21	4	25
Lack of parental education regarding importance of NHS	33	28	28	16	3	19
Referrals from lower-level services	0	0	4	2	0	0
Quiet areas to conduct screening	0	0	2	1	0	0

**Total**	**118**	**100**	**180**	**100**	**16**	**100**

NHS, newborn hearing screening; NGO, non-governmental organisation.

Sector-specific patterns were evident. In the private sector, a key concern was the lack of medical aid coverage for hearing screening, reported by 18% (*n* = 21) of respondents. Other notable issues included time constraints (14%; *n* = 16) and non-attendance for initial screening appointments (13%; *n* = 15). In contrast, public sector respondents highlighted operational barriers, including staff shortages (16%; *n* = 28), time constraints (16%; *n* = 29) and malfunctioning or uncalibrated equipment (11% each; *n* = 20 and *n* = 19, respectively). Although NGO sector participants represented a smaller sample, similar operational challenges were reported, including staff shortages (19%; *n* = 3), time constraints (13%; *n* = 2) and a lack of funding (13%; *n* = 2). Issues such as the absence of quiet screening areas and poor referral systems from lower-level services were only mentioned in the public sector, albeit infrequently.

### Reasons for non-implementation of newborn hearing screening programmes

Twenty-five participants from the private, public and NGO sectors reported that they do not conduct NHS. The most common reasons cited were the high cost of equipment (41%; *n* = 9), staff shortages in the public sector (32%; *n* = 7), and broken equipment, also predominantly in the public sector (14%; *n* = 3).

### Referral practices in the absence of newborn hearing screening services

All audiologists in the private sector who do not offer newborn hearing screening (*n* = 7) reported referring infants to colleagues who do provide screening services. In the public and NGO sectors (*n* = 18), infants are typically referred to the nearest healthcare facility where NHS is available. In most cases, the referral destinations were either district hospitals (22%; *n* = 4) or tertiary-level hospitals (22%; *n* = 4).

## Discussion

This study provides a comprehensive overview of the status, personnel, equipment availability and challenges associated with NHS services across public, private and NGO health sectors in South Africa. The findings highlight both progress and persistent inequities in the implementation of early hearing detection and intervention (EHDI) services.

### Geographic and sectoral disparities in newborn hearing screening availability

The availability of NHS services remains unevenly distributed across South African provinces, with a significant concentration in urban and economically advantaged provinces such as Gauteng and the Western Cape. These two provinces alone accounted for more than half (56%) of all reported NHS services. In contrast, provinces such as the North West, Limpopo and Mpumalanga are critically underserved, particularly in the private and NGO sectors. This urban-rural divide in NHS coverage reflects broader systemic inequities in healthcare access and human resource distribution (Pillay et al., 2020) and aligns with previous research documenting regional disparities in EHDI implementation across South Africa (Kgare & Joubert, 2024; Khoza-Shangase et al., 2017). Although the public sector provided the greatest number of NHS services overall (49%), these services were predominantly offered at tertiary-level hospitals, with limited integration at primary and community healthcare settings. This centralisation of services presents a significant barrier to timely detection, particularly in rural and underserved communities where access to tertiary care is limited (Olusanya et al., 2004; WHO, 2021a). Decentralisation and integration of NHS into lower-level health facilities remain critical to improving service coverage, accessibility and equity (Kanji, 2022; Kgare & Joubert, 2024; Petrocchi-Bartal & Khoza-Shangase, 2016). Notably, the findings also highlight the potential and growing contribution of non-governmental organisations (NGOs) and public–private partnerships in addressing service gaps, particularly in provinces where state-provided healthcare infrastructure is lacking. Although NGOs accounted for only a small proportion of services, they reported high relative levels of equipment availability, suggesting that such entities can provide well-resourced, targeted interventions. Similarly, public–private partnerships have demonstrated the capacity to extend NHS services into underserved settings by leveraging shared resources and expanded workforce capacity (Joudyian et al., 2021; Kgare & Joubert, 2024). These collaborative models should be further explored and scaled to enhance reach and sustainability, especially in support of achieving the WHO’s 2030 target of increasing NIHS coverage by 20% (WHO, 2021a).

### Equipment availability and technological gaps

Availability of screening equipment was generally high across all sectors, particularly for AABR and OAE. This aligns with previous findings that these tools are considered the gold standard for objective screening in newborns and infants (Kanji, 2021a; Olusanya et al., 2014). NGOs, while representing a smaller portion of the sample, demonstrated a notably high relative availability of screening equipment, suggesting targeted investment and external donor funding that enable well-equipped service delivery even in otherwise under-resourced contexts. Despite relatively good access to conventional screening equipment, the use of mHealth technologies was limited. Only 4% (*n* = 2) of public sector facilities reported use of mHealth tools, with no reported use in the private or NGO sectors. This represents a significant missed opportunity, given the mounting global interest in leveraging mHealth for expanding healthcare access in low- and middle-income countries, particularly where geographic and infrastructural barriers exist (Swanepoel & Clark, 2019; WHO, 2021a). Evidence from South Africa and other resource constrained contexts has shown that smartphone-based hearing screening applications can deliver accurate results while significantly lowering costs and logistical demands (Madzivhandila et al., 2024; Swanepoel & Clark, 2019). Given that mHealth screening devices are relatively new to the South African context, further implementation research may be necessary to inform national scale-up efforts, particularly in rural and primary care settings (Madzivhandila et al., 2024).

### Personnel and task-shifting practices

Most NHS services in all sectors were conducted by audiologists, affirming their central role in the implementation and oversight of EHDI programmes. However, given the shortage and uneven distribution of audiologists in South Africa, particularly in rural provinces and within the public health system (Pillay et al., 2020), reliance solely on audiologists is not sustainable for achieving universal coverage. The inclusion of trained non-audiologist screeners, particularly in the NGO sector, suggests a gradual shift toward task-sharing models. These models, when appropriately supervised and supported, can enhance service delivery in settings with a limited audiology workforce (Eksteen et al., 2019; Kgare & Joubert, 2024; Suen et al., 2019; WHO, 2020). Encouragingly, all non-audiologist screeners in this study were trained and supervised by audiologists, which aligns with global recommendations for maintaining screening quality within task-shifting frameworks (JCIH, 2019).

### Challenges in newborn hearing screening implementation

Participants across all sectors reported a range of challenges affecting the successful implementation of NHS. The two commonly cited issues, poor parental awareness of NHS and failure to return for follow-up, highlight the persistent gap in caregiver education and underscore the urgent need for improved caregiver education. These challenges have been consistently identified in both local and international research as major impediments to effective early hearing detection and intervention (Das et al., 2020; Ehlert & Coetzer, 2020). Caregiver knowledge, beliefs and perceived urgency around hearing loss are well-established determinants of screening follow-up rates. Without targeted public awareness campaigns, many families may not understand the long-term developmental implications of missed or delayed diagnosis, nor the necessity of follow-up appointments when initial screening results are inconclusive or indicate possible hearing loss (Das et al., 2020). In resource-constrained settings such as South Africa, operational barriers further exacerbate these challenges. In the public and NGO sectors, participants reported chronic staff shortages and significant time constraints. These findings are consistent with earlier studies that have documented insufficient audiology posts in the public sector, compounded by hiring freezes and budgetary austerity (Naidoo & Khan, 2022; Pillay et al., 2020). A lack of equipment was also reported, consistent with findings of a recent study that explored the availability of hearing screening equipment at public hospitals (Bhamjee et al., 2022). In contrast, the private sector, while generally better resourced in terms of staffing and equipment, faces unique economic constraints. One prominent barrier identified was the lack of medical aid coverage for NHS services, which limits uptake, a barrier that has been previously highlighted (Das et al., 2020; Theunissen & Swanepoel, 2008). These challenges collectively illustrate the need for sector-specific strategies, including resource investment, advocacy for funding mechanisms, and integration of screening into existing maternal and child health workflows.

### Barriers to implementation and referral practices

Among participants who did not conduct NHS, the most frequently reported barriers were high equipment costs, a lack of staff and broken equipment, particularly in the public sector. These findings are consistent with research in South Africa, which has highlighted capital investment limitations and the impact of austerity-driven hiring freezes on the provision of essential services (Khoza-Shangase, 2021, 2025; Naidoo & Khan, 2022). Despite these barriers, referral practices in the absence of on-site NHS services were generally appropriate. In the private sector, audiologists who did not offer NHS reported referring infants to colleagues within their professional networks. In the public and NGO sectors, referrals were made to the nearest district or tertiary hospitals where NHS services were available. This practice aligns with standard EHDI protocols that emphasise prompt referral following risk identification or failed screening (JCIH, 2019). However, reliance on external referral systems is problematic when geographic proximity, transport availability or service capacity are lacking, conditions commonly encountered in rural and under-resourced communities (Kgare & Joubert, 2024). Strengthening referral pathways and ensuring continuity of care remain vital components of a comprehensive EHDI system (Khoza-Shangase, 2025).

### Study limitations

The study has several limitations that should be acknowledged. Firstly, the cross-sectional survey design and recruitment through professional organisations may have led to selection bias, with potentially lower uptake from audiologists in under-resourced or rural areas who may have limited access to professional networks or online platforms. This may have resulted in under-representation of perspectives from critically underserved provinces. Secondly, the data were collected between March 2022 and July 2022 and therefore do not capture any NHS initiatives or policy developments implemented in the subsequent period. Given the rapidly evolving landscape of hearing healthcare in South Africa, more recent developments such as expanded mHealth implementations or new public–private partnerships are not reflected in these findings. Thirdly, the self-reported nature of the survey data may introduce response bias, particularly regarding equipment availability and service coverage.

## Conclusion and implications

This study provides updated, nationally representative insights into the current status and practices of NHS in South Africa across public, private and NGO sectors. While notable progress has been made, particularly in equipment availability and the growing role of NGOs, critical geographic, structural and systemic barriers persist. Newborn hearing screening services remain disproportionately concentrated in urban centres and tertiary-level hospitals, limiting access for infants in rural or underserved communities. In addition, the underutilisation of cost-effective innovations such as mHealth technologies and persistent workforce shortages further constrain the scalability and sustainability of NHS programmes. Task-shifting strategies involving trained non-audiologist screeners appear promising, especially when appropriately supervised by audiologists, and should be expanded in line with global recommendations for EHDI. To advance equitable NHS coverage nationally, several policy and practice implications emerge. Firstly, there is an urgent need for strategic decentralisation of services to primary and community health facilities, particularly in underserved provinces. Secondly, increased investment in human resources, equipment maintenance and the integration of NHS into existing maternal and child health workflows are essential to overcoming implementation challenges. Thirdly, targeted public education campaigns are required to raise awareness among caregivers regarding the importance of early hearing detection and follow-up. Finally, collaborative approaches, including NGO engagement, intersectoral partnerships, and public–private initiatives, must be leveraged to bridge service gaps, especially in areas where state capacity is limited. These measures will be critical to achieving the WHO’s 2030 goal of expanding newborn and infant hearing screening coverage by at least 20% and ensuring that all South African children receive timely and equitable access to hearing healthcare.
